# Supplementary material for: Patterns of Intron Gain and Loss in Fungi
Source: PLoS Biol. 2004 Nov 30;2(12):e422. doi: 10.1371/journal.pbio.0020422 (PMC532390; doi:10.1371/journal.pbio.0020422)
Supplement: Table S1 — Also available at http://genes.mit.edu/NielsenEtAl/. (4.3 MB ZIP). [file pbio.0020422.st001.zip › NielsenEtAl/html/402.html]

AN0809.1.NCU02103.1.MG04652.1.FG00916.1


```
 CLUSTAL W (1.82) Multiple Sequence Alignments - Introns Inserted


Sequence 1: NCU02103.1	1752 aa
Sequence 2: MG04652.1	1745 aa
Sequence 3: FG00916.1	1758 aa
Sequence 4: AN0809.1	1745 aa
Alignment Length: 1801 aa
Number Identitical Residues: 933 aa
Alignment Score (without introns) 43818


MG04652.1 	MANLIFAPSSAPLKTIEEIQFGVLAPEEIKNMSVVHVQYPDTM~DETRTKPREGGLNDPL
NCU02103.1	MANLYFAHSSAPLRTIKEIQFGLLSPEEIKGMSVAHIVYPETM~DETRTKPRDGGLNDPL
FG00916.1 	MANLYFTHSSAPLKTVEEIQFGLMSPEEIKNMSVCHIVYPETM~DENKTKPRDGGLNDLL
AN0809.1  	MSNVYFPYSKAPLRTIAEIQFGLFSPEEIKRMSVVHVEYPETM0DDQRQRPRTKGLNDPR
          	*:*: *. *.***:*: *****:::***** *** *: **:** *: : :**  ****  

MG04652.1 	LGTTDRQYKCKTCNNDMNNCPGHFGHIELAKPVYHPG~FINKTKKILEM0N---------
NCU02103.1	LGSVDRQFKCKTCGENMSECPGHFGHIELARPVYHPG~FIKRVKKMLEI~VCHNCSKVLA
FG00916.1 	LGSIDRSFRCKTCTQAMGECPGHFGHIELAKPVYHPG~FIKKVKKILEI~VCHNCSKVLA
AN0809.1  	LGTIDRQWNCETCEEGQKECPGHFGHIELATPVFHIG1FLTKIKKLLET~VCHNCGKIKA
          	**: **.:.*:** :   :*********** **:* * *:.: **:**   . ....  :

MG04652.1 	--~NDEQFAQALRLRDPKARFAMVWKACKGKKVCEIETGGKDEKDSID-TATGIEKVP-H
NCU02103.1	DR0NDKEFAAAMRIRDPKVRFNRVWAVCKKKRRCENEVVKKSDDDEFNPLADAKGELPGH
FG00916.1 	DS0RDPEFAAAINTRDPKVRFHRVWEACKKKKRCENDEPKQKDDEFAATLKTG--PLEGH
AN0809.1  	NT0SDPKFLDALRIRDPKRRFDHIWRLSKDILICEADPPPEDDE----YGKESSKPVRLH
          	.   * :*  *:. **** **  :*  .*    ** :   :.:.        .   :  *

MG04652.1 	GGCGSTHPDIRKKALQLYAKVEEA---REE-GMKKEVKD-KLITPEQAHHILKHIPEDDL
NCU02103.1	GGCGNDHPTVRQQALTLYAQFDTK---DEE-GVKSKEK--KIITPEMALNILHNMTESDM
FG00916.1 	GGCGNVQPAVRQGALQLKAAFEVT---VDEDGQKTKKKETIPITPETAHGILRRISEEDL
AN0809.1  	GGCGNAQPTVRKEGITLVGTWKPSKSMMDEMDMQQPEK--KIITPQMALNIFRNISHEDV
          	****. :* :*: .: * .  .  .:  :* . :   *    ***: *  *::.:...*:

MG04652.1 	WKMGLNKDYARPEWLIVTVLPVPPPPVRPSISVDGTSQGMRSEDDLTYKLGDIIRANGNV
NCU02103.1	IDIGINISQARPEWMIITVLPVPPPPVRPSISMDGTGTGMRNEDDLTYKLGDIIRANGNV
FG00916.1 	VNMGLNSDYARPEWMVLTVLPVPPPPVRPSISMDGTGTGMRNEDDLTYKLGDIIRANGNV
AN0809.1  	RIMGLSNDYARPEWMILTVLPVPPPPVRPSVVVGGSTSGQRGEDDLTYKLAEIVRANQNV
          	  :*:. . *****:::*************: :.*:  * *.********.:*:*** **

MG04652.1 	KQAHAEGAPNHICTEFEELLQYHVATYMDNDIASIPRSLQKSGRPIKSIRARLKGKEGRL
NCU02103.1	RQAIQEGSPAHIINDFENLLQYHVATYMDNDIAGQPQALQKSGRPVKAIRARLKGKEGRL
FG00916.1 	KQAIREGSPQHIARDFEELLQYHVATYMDNDIAGQPRALQKSGRPVKAIRARLKGKEGRL
AN0809.1  	QRCEQEGAPEHVVREFESLLQYHIATYMDNDIAGQPKAMQKSNRPVKAIRSRLKGKEGRL
          	::.  **:* *:  :**.*****:*********. *:::***.**:*:**:*********

MG04652.1 	RGNLMGKRVDFSARTVITGDANISLHEVGVPRSIARTLTYPETVTPHNIDRLHQYVVNGP
NCU02103.1	RGNLMGKRVDFSARTVITGDANISLDEVGVPRSIARTLTYPETVTPYNIGKLTQYVQNGP
FG00916.1 	RGNLMGKRVDFSARTVITGDANLSLHEVGVPRSIARTLTYPETVTPYNIEKLHQLVENGP
AN0809.1  	RQNLMGKRVDFSARTVITGDPNLSLDEVGVPKSIARTLTYPEVVTPYNIEKLQQLVMNGP
          	* ******************.*:**.*****:**********.***:** :* * * ***

MG04652.1 	NEHPGAKYIVRTDGTRIDLRHVKNRGSTQLEYGQKVERHLLTGDYIIFNRQPSLHKESMM
NCU02103.1	NEHPGAKYVIRSDGSRVDLRHQRRTGPLQLEYGWKVERHLIDGDYIIFNRQPSLHKESMM
FG00916.1 	NEHPGAQYVVRPDGSRIDLRHHRRAGAISLEYGWKVERHLMTGDYIIFNRQPSLHKESMM
AN0809.1  	NEHPGARYIVRDNGERIDLRHAKRAGGQQLLYGWKVERHLMDGDVILFNRQPSLHKESMM
          	******:*::* :* *:**** :. *  .* ** ******: ** *:*************

MG04652.1 	GHQVRVMPYSTFRLNLSVTSPYNADFDGDEMNLHVPQTEETRAEVKELCMVPLNIVSPQR
NCU02103.1	GHRVKVMPYSTFRLNLSVTSPYNADFDGDEMNLHVPQTEETRAEVKELCMVPLNIVSPQR
FG00916.1 	GHRVRVMPYSTFRLNLSVTSPYNADFDGDEMNLHVPQSEETRAEVKELCLVPLNIVSPQK
AN0809.1  	GHRVRVMPYSTFRLNLSVTSPYNADFDGDEMNLHVPQSEESRAELQQLALVPQNIVSPQR
          	**:*:********************************:**:***:::*.:** ******:

MG04652.1 	NSPLMGIVQDTLAGTYKLCRRDVFLTKEAVMDIMLWVPDWDGIIPQPAILRPRPRWTGKQ
NCU02103.1	NGPLMGIVQDTLAGAYKLCRRDVFLTKEEVMNIMLWVPEWDGIIPVPAILKPRPRWTGKQ
FG00916.1 	NGPLMGIVQDSLAGAYKMCRRDVFLTKEQIMNCMLWVPNWDGVIPQPAIYKPRPRWTGKQ
AN0809.1  	NGPLMGIVQDTLCGIYKICRRDVFLTKEQVMNIMMWVPDWDGVIPPPAIFKPRPRWTGKQ
          	*.********:*.* **:********** :*: *:***:***:** *** :*********

MG04652.1 	IISMVIPDFVTTHQGG------DWN---PLNDDALLIQSGELMFGLLTKKNVGAASGGII
NCU02103.1	IISMVVPKIINLKNID------EDDGECPLKDKGILIQQGEVIFGLMTKKIVGAASGGIV
FG00916.1 	LISMVIPKEVTIFNGT------DSGESAPLKDEGLLIQAGQLMYGLLTKKSVGASAGGIV
AN0809.1  	MISMVFPSGLNLMRTDSKGAAPSSEKYSPLQDGNVLIHEGQLMYGMLNKKIVGASGGGVI
          	:****.*. :.  .  :..::..    .**:*  :**: *::::*::.** ***:.**::

MG04652.1 	HICYNEQGPARAMAFLNGCQRVVNYWLLHNGHSIGIGDTIPDPVTIEKIQAHINEQKAEV
NCU02103.1	HLCYNELGPSGAMDFLNGCQRVVNYWLLHNGHSIGIGDTIPDAATIAKVQTHIDDAKAEV
FG00916.1 	HISYNELGPEGAMAFLNGVQQVVTYWLLQNGHSIGIGDTIPDAVTIAKVQAHIDDEKAEV
AN0809.1  	HIIFNEYGADAAVAFFNGAQAIVNYWLLHNGFSIGIGDTIPNDQTIQAIEECVRKRKLEV
          	*: :** *.  *: *:** * :*.****:**.*********:  **  ::  : . * **

MG04652.1 	AELTDLATTNRLEALPGMNIRETFESKVSKALNTAREKAGTTTEKSLKDINNAVTMSRSG
NCU02103.1	KQLTDMATNNQLEPLPGMNIRETFENKVSKALNTARDKAGTTTESSLKDINNAVTMALSG
FG00916.1 	ARLTAMATANELEALPGMNVRATFENKVSMALNQARDKAGTTTQKSLKDSNNAVTMASSG
AN0809.1  	EEITATATQNKLEPLPGMNVRETFESKVSVALNTARDEAGTATEKSLKDLNNAVQMSRSG
          	 .:*  ** *.**.*****:* ***.*** *** **::***:*:.**** **** *: **

MG04652.1 	SKGSSINISQMTALVGQQIVEGKRIPFGFKYRTLPHFTKDDFSPEARGFVENSYLRGLTP
NCU02103.1	SKGSSINISQMTALVGQQIVEGKRIPFGFKYRTLPHFTKDDYSPEARGFVENSYLRGLTP
FG00916.1 	SKGSSINISQMTALVGQQIVEGKRIPFGFKYRTLPHFTKDDYSPEARGFVENSYLRGLTP
AN0809.1  	SKGSIINISQMTAVVGQQMVEGKRIAFGFKYRTLPHFTKDDYSPESRGFVENSYLRGLTP
          	**** ********:****:******.***************:***:**************

MG04652.1 	SEFFFHAMAGREGLIDTAVKTAETGYIQRRLVKALEDVAAKYDGTVRNSLGDIIQFIYGE
NCU02103.1	SEFFFHAMAGREGLIDTAVKTAETGYIQRRLVKALEDSAAQYDGTVRNSLGDIIQFVYGE
FG00916.1 	SEFFFHAMAGREGLIDTAVKTAETGYIQRRLVKALEDLSARYDGTVRNSLGDIVQFLYGE
AN0809.1  	TEFFFHAMAGREGLIDTAVKTAETGYIQRKLVKALEEVMVKYDGTVRNSLGDIIQFIYGE
          	:****************************:******:  .:************:**:***

MG04652.1 	DGLDGMAIEKQKMDHLNISNGKFDGMYKLDLMTATRPR-ELNALEYGNEIFGDPAVQQLL
NCU02103.1	DGLDGIAIENQPVDHFTISNKAFDKRYRLDVMDEEVSSGELEALEYGKELASDPTVQELL
FG00916.1 	DGLDAMIIENQKLGILNMSNTAFEKKYRLDLANP--PEWFKHDYEFGNELTGDRPSMALL
AN0809.1  	DGLDGAHIEHQKVDILTCSDEKFKERFRIDLMDP-ALSLGPDVLEQANEIAGDVEVQRYL
          	****.  **:* :. :. *:  *.  :::*:          .  * .:*: .*      *

MG04652.1 	DEEYDAIKADRQLIREINYKK-KDDNDMQLPLNVARVIENAKKLFKIDDAQRSDLKPQDV
NCU02103.1	DQEYEQLSVDRHMLREIQRTKGNNDTKMQLPLNIIRIIDTAKKLFKVDETARSDLTPKDV
FG00916.1 	DEEWERLVHDRTRIRAINRAK-GNEEMMQLPLNITRIIESAKRVFNVKANDRSNLRPSDV
AN0809.1  	DQEWEELLKAREVVRHVAKGT---EDSMPLPINIQRILEMARTTFRIREGTMSDLHPAEV
          	*:*:: :   *  :* :   .   :  * **:*: *::: *:  *.:     *:* * :*

MG04652.1 	IPVVKGFLERMVVVPGDDPISKEANYNSSILFKAQLRSRLAFKRLAVRERLNRIAFDHVI
NCU02103.1	IPAVKAMLDRMIVVRGDDPISKEADYNATILFKAQLRSRLAFKRLAVQDRINKLAFNHIL
FG00916.1 	IPAVQNMLDNMKIVRGTDPISLEADANASILFKGLLRSRLAFKEVVKEHRLNRLAFDHIL
AN0809.1  	IPQVQSLLDRLVIVRGDDPISKEAQESATMLFKAQLRSRLAFKRLVTQYSMNKLAFQHVL
          	** *: :*:.: :* * **** **: .:::***. ********.:. .  :*::**:*::

MG04652.1 	GEMESRWQRSFVSPGEMVGVLAAQSIGEPATQMTLNTFHFTGISSKNVTLGVPRLKEILN
NCU02103.1	GELENRWSRSMVSPGEMVGVLAAQSIGEPATQMTLNTFHFAGVSSKNVTLGVPRLKEILN
FG00916.1 	GELQNRWDRAFVNPGEMVGVLAAQSIGEPATQMTLNTFHFAGVSSKNVTLGVPRLKEILN
AN0809.1  	GAIESRFAKAAAAPGEMVGVLAAQSIGEPATQMTLNTFHFAGVSSKNVTLGVPRLKEILN
          	* ::.*: :: . ***************************:*:*****************

MG04652.1 	VSAEIKTPGMVVRLDPKMYPDFKQENAKTLRSLVEHTNLRSVTAMTEIYYDPEIQTTTID
NCU02103.1	VASNIKTPSMMVYLDSKAA---TQEEAKKMRSAVEHTSLRSVTAVTEIYYDPDITSTNIP
FG00916.1 	LAKNIKTPSMAVYLNTPLA---KQEQAKKLRSMVEYTNLRSVTSVTEIYYDPNVTETNIP
AN0809.1  	VATNIKTPSMTVYQEPERAH--DKEGAKQLRSAVEYTNLRSITEATEIYYDPDIQTTVIE
          	:: :****.* *  :.       :* ** :** **:*.***:*  *******::  * * 

MG04652.1 	EDADMLEGHYLIPEEHQDEPS---NQSRWLLRLTLDRQKMLDKNLHIEDVAASIKGAYAS
NCU02103.1	EDYDLVESYFLIPDQSGDEPDPIENQSRWLLRITLDRQKMLDKGLRVEDVAHRIKEVYKK
FG00916.1 	EDLDMVESYYMIPDESVDPTA---NQSRWLLRITLDRQKLLDKEIKIDDVAQRIKEEYPT
AN0809.1  	NDRDMVESYFIIPEDVNDDSS---RQSKWLLRIILSRAKLLDKGLTVQDVATKIKQAYPR
          	:* *::*.:::**::  * .    .**:****: *.* *:*** : ::***  **  *  

MG04652.1 	DLVVVHSDNNADEQVIRMRHVIKGDEEDKDG-RNIESDQILKRLEEHMLDGLTLRGIKGI
NCU02103.1	DVAVVFSDNNAEEMVIRIRVIKADDDKDEDGNKIIEDDVMLKRLEKHLLDGCTLRGVEGI
FG00916.1 	DLAVIFSDNNADEQVIRIRTIHTGDKDDDGDGGRMEDDVMLKRLEAHLLDTLTLRGVPGI
AN0809.1  	DIAVIFSDNNADEQVIRIRQIQDYKEDEDDD--DIEYDVTLKKLEQHLLDTLNLRGVPGV
          	*:.*:.*****:* ***:* :   ...:...   :* *  **:** *:**  .***: *:

MG04652.1 	ERAFLNKDTRLVVKPDGSLLNSKDAPECEEWYLDTQGTSLRQVLCVDGVDSVRTLTNDLY
NCU02103.1	ERAFLNKGVKLIEAKDGSMKHSKVDDECQEWYLDTQGTSLREVLTVEGVDTKRTTTNDLW
FG00916.1 	ERAFLTKGTKLSEDDDGALLAIKDDPRCTQWYLDTSGTALRDVLAVDGVDATRTYTNDLY
AN0809.1  	ERAFINEKKKIRVQEDGALTDEQSDPLCREWILETSGSSLAEVLAIPGVDATRTYSNQFI
          	****:.:  ::    **::   :    * :* *:*.*::* :** : ***: ** :*:: 

MG04652.1 	QIVDLLGIEAARTALLGELTQVLAFDGSYVNHRHLALLVDVMTYRGSIAAVTRHGINRAD
NCU02103.1	QVVDVFGIEGARAALMHELTAVLAFDGSYVNHRHLALLCDVMTYRGSIAAVTRHGINRAD
FG00916.1 	QIVEVFGIEAARSALAKELTNVLAFDGSYVNHRHIALLVDVMTYRGVISAVTRHGINRAD
AN0809.1  	EVFEVFGIEAARAAVLREFTVVLSFDGSYVNHRHLALLVDVMTMRGYLTPVTRHGINRAD
          	::.:::***.**:*:  *:* **:**********:*** **** ** ::.**********

MG04652.1 	TGALMRCSFEETVEILLEAAAIGELDDCRGISENVMLGQLAPMGTGAFEVLLDPKMLETV
NCU02103.1	TGALMRCSFEETVEILLEAAAVGELDDCRGISENVMLGQMAPMGTGAFDVFLDPKMLETV
FG00916.1 	TGALMRCSFEETVEILLEAAATGELDDCRGISENVMLGQMAPMGTGNFDVLLDPKMLETV
AN0809.1  	NGALMRCSFEETVEILLEAAAFGELDDCRGVSENLILGQMAPAGTGEFDIYLDQNMLNTV
          	.******************** ********:***::***:** *** *:: ** :**:**

MG04652.1 	VSNNARMGLMPGLTVKGSSA-EGAATPYDTGSPMGDSGYNLGMASPTMGNFSPIMSGGSD
NCU02103.1	ISDNSRMGLMPGMTVKGSQL-DGAATPYDTGSPMADSGF-LGSYSPTMGNFSPIQGAGSD
FG00916.1 	ISDNSRMGLMAGMPTKSGDI-EGAATPYDTGSPMADSGY-LSMSSPAAGNFSPIQGAGSD
AN0809.1  	VSNNARYGVMGALGAKDAIISDGAATQYDTGSPMQESAY-IGTPDPDS-NFSPIRQAGAE
          	:*:*:* *:* .: .*..  ::**** ******* :*.: :.  .*   *****  .*::

MG04652.1 	TPT--GFATEYGG-GSLGSMNPYGSGTSPR-PTSPFSTS-PTSPFSAGYGSYSPTSPMAG
NCU02103.1	SPTNNGFGTDYGGVGGYGSGTSYSSATSPR-ATSPFT---PTSPFS--YGGYSPSSPAAG
FG00916.1 	TPA--GFDTVYGG-GSVGFGGTTPMSPYNRGAASPFSSTSPTSPFS-----YSPTSPNMG
AN0809.1  	TPGG---FTEYQPSGGFGGFSPAATSPAGYSPSSPFSAN-PTSPGYSPTSSYSPTSPGMG
          	:* .    * *   *. *   .   ..   ..:***::. ****  :  ..***:**  *

MG04652.1 	YSPTSPMMDGG---RYGASSPSFSPSSPSFQPTSPIMRHGAGDSMGYSPTSPTYQPGSPT
NCU02103.1	YSPSSPLLDSA---GRFASSPQFSPSSPSFSPTSPMLRPGSPTSPSYSPTSPSYSPASPA
FG00916.1 	YSPTSPLIDGGGMGRYGPTSPSFSPSSPSFSPTSPMLRPTSPASPSYSPTSPSYSPTSPS
AN0809.1  	ITSPRFMTSPG----FSPASPSFAPTSPAYSPTSPAYGQASPTSPSYSPTSPGFSPTSP-
          	 :..  : . .      .:**.*:*:**::.****     :  * .****** :.* ** 

MG04652.1 	GGDNHYSPTSPAQFGASPTSPLYSPASPSFSPGG-------------ATSPMYYVGSPQH
NCU02103.1	AAR--YSPTSPAQQ-FSPTSPSYSPTSPSYSPASPAFQ---------ATSPSYSPASP-T
FG00916.1 	SPR-HYSPTSPAQF-NSPTSPSYSPASPNYSPTSPNVHGA-----GGPTSPSYSPASP-S
AN0809.1  	----NYSPTSPSFS---PTSPAFSPTSPSYSPTSPAIGGARHLSPTSPTSPKYTPTSP-G
          	     ******:     **** :**:**.:** ..   .:   :. ..*** *   **  

MG04652.1 	ASPTSPNAHYSPTSPSFQRSP-AQASPTSPGPYSPT0SPSFSPR~SPGRNNRSGGQDSY2
NCU02103.1	WSPTSPDA-YSPTSPSFQRSPGQQLSPTSPTGYSPT~SPQYSPR~TPGRNNSGGNGDQ-~
FG00916.1 	WSPTSPEA-YSPTSPTFQRSPTTQQSPTSPS-YSPT~SPAFSPR~TPGPGTSG---NQY2
AN0809.1  	WSPTSPQT-YSPTSPNFAGSPTSPGGPTSPG-YSPT~SPAFSPT2SPRQ----------~
          	 *****:: ******.*  **    .****  **** ** :**  :*             

MG04652.1 	SPTSPSHD
NCU02103.1	--------
FG00916.1 	SPNSPSND
AN0809.1  	--------
          	
```
